# Supplementary figures and images for: Stabilized Astaxanthin Nanoparticles Developed Using Flash Nanoprecipitation to Improve Oral Bioavailability and Hepatoprotective Effects
Source: Pharmaceutics. 2023 Oct 31;15(11):2562. doi: 10.3390/pharmaceutics15112562 (PMC10675309; doi:10.3390/pharmaceutics15112562)

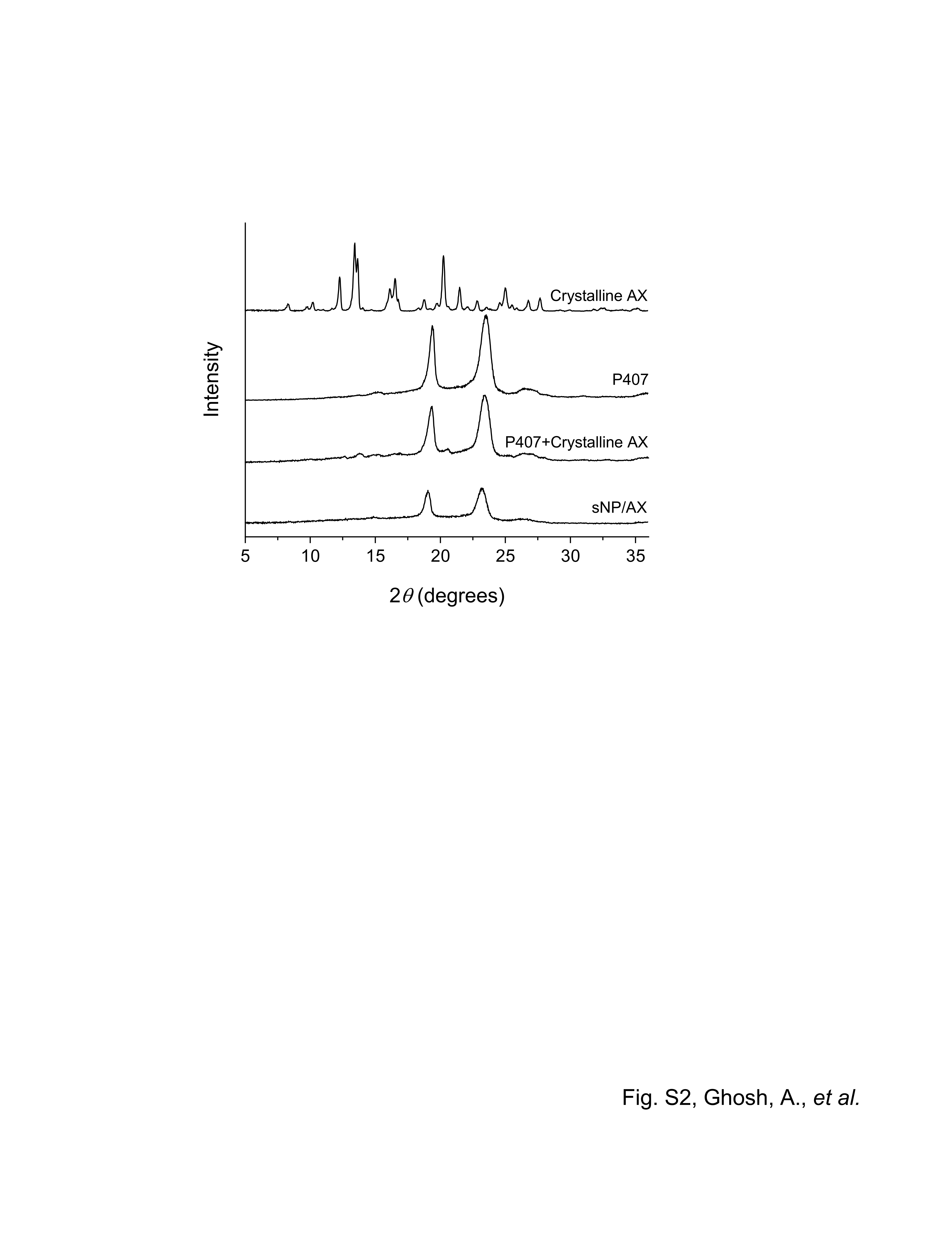

Supplement: Supplementary file 1 [file pharmaceutics-15-02562-s001.zip › Fig. S2_XRPD.tif]
